# Is Compulsive Sexual Behavior Different in Women Compared to Men?

**DOI:** 10.3390/jcm10153205

**Published:** 2021-07-21

**Authors:** Laura I. Kürbitz, Peer Briken

**Affiliations:** Institute for Sex Research, Sexual Medicine and Forensic Psychiatry, University Medical Center Hamburg-Eppendorf, Martinistr. 52, 20246 Hamburg, Germany; briken@uke.de

**Keywords:** CSBD, hypersexuality, sex addiction, gender, female, Compulsive Sexual Behavior

## Abstract

The inclusion of Compulsive Sexual Behavior Disorder (CSBD) in the ICD-11 has sparked research interest on this topic in recent years. This review aims to investigate gender differences in Compulsive Sexual Behavior (CBD) and persons with CSBD. While impulsivity and psychiatric comorbidity play a role in persons with CSBD regardless of gender, some gender differences can be identified. CSBD is more prevalent in men, with a higher rate of reported sexual behaviors and higher scores on questionnaires measuring CSBD related symptoms. Neuroticism and stress vulnerability seem to play a more important role in the symptomatology of CSBD in women. While it seems plausible that childhood adversities play a role in the development of CSB, the manner with respect to how these adversities affect men and women differently is still to be explored. More clinical studies including the female CSBD population are required to infer clinical implications.

## 1. Introduction

Compulsive Sexual Behavior Disorder (CSBD) is a new diagnostic category that was first established in the ICD-11 [1]. While the diagnosis is new, it is a new label for an old phenomenon. Since more than 100 years ago, researchers have described people with excessive sexual behavior that causes trouble in other life areas [2]. It was hypothesized that people with CSBD (or “hypersexual disorder” or “sex addiction”) use sex as a fill-in for other behaviors (e.g., reduction in tension) and sexual behavior causes significant distress or impairment [3]. There has been a lot of debate on whether CSBD is a definable diagnosis and in what category it should fall under. Researchers and clinicians classify it as a behavioral addiction [4], an obsessive-compulsive or impulse control disorder [5] or simply a symptom of other underlying mental health problems [6]. CSBD is now described by the following symptoms: A strong urge to engage in sexual behavior, the use of sexual behaviors to cope with adverse emotional states, having tried to control these urges and experienced failure to do so in the past and engaging in sexual behavior repeatedly in disregard of adverse consequences [2].

Patients affected with CSBD continue with their sexual behavior even though they derive no or only little satisfaction from it [1]. The sexual behavior interferes with daily activities such as family, social and career development. While people with CSBD are often suffering intensely, some of them are also suffering quietly. Studies have shown that people with CSBD are often stigmatized and experience shame and guilt for their behavior, which in turn drives them to hide it [7]. Even people who are in treatment for other psychological problems report that they do not mention their sexual behavior in therapy or treatment [6].

When talking about CSBD, it is important to distinguish between people having a high sexual drive and patients suffering from CSBD. Individuals with a high sex drive should not be pathologized if the high sex drive does not result in suffering [2] or if the suffering is only mediated by sex-negative social norms (e.g., restrictive and maybe religious thoughts about sex and sex drive) [8].

As researchers and clinicians are also influenced by biases, CSBD was mostly regarded as a male problem in the past. Especially with respect to women, shame seems to be induced in talking about Compulsive Sexual Behavior (CSB) [9]. The research focused on “typical female” sexual dysfunctions such as low libido and pain, which were studied intensely [10]. This resulted in the status quo: CSBD is still not well researched and the little research we have has been conducted mainly in men. Another reason for this disparity is possibly that CSBD might have different manifestations in women compared to men. This results in barriers in the treatment of female individuals with CSBD. These barriers occur in the individual (e.g., not admitting to oneself to having a sexual problem), in the society (different societal norms for men and women), in research (CSB is under researched in women) and treatment (e.g., stigma) [6].

Even though the behavior is better studied in men, epidemiological data show that women frequently do report out of control sexual behavior as well. Epidemiological data suggests that as much as 7% of women report that they had the feeling in the past that their sexual behavior was out of control [11,12,13]. However, a lot of women do not seek out treatment due to shame [6].

Contrary to this line of argument [6], Werner et al. showed via cluster analysis that the symptoms of men and women are not as dissimilar as suspected [14]. Women as well as men use pornography and sexual encounters to live out their compulsive sexual behavior. They do have dissimilar fears though as women do fear pain and bodily harm more than men [15].

Even though some gender differences have been reported in the past, a thorough and comprehensive overview of gender differences in CSBD is lacking. The aim of this review is to identify differences regarding symptoms and psychiatric comorbidities between men and women.

## 2. Materials and Methods

In order to conduct our review, we did a conducted search of PubMed, Psyndex, PsycInfo, PsycArticles and Web of Science according to PRISMA statement with the following mesh terms.

“Compulsive Sexual Behavior Disorder” OR “Compulsive Sexual Behavior” OR “Hypersexuality” OR “Sexual Addiction” OR “out of control sexual behavior” OR “Hypersexual Disorder” OR “Hypersexual” OR “Compulsive Sex”.

The search was roughly narrowed down to the last 20 years (2000–June 2021) because the concept of CSBD has changed considerably in the last decades. Last day of retrieval was 17 Jun 2021. With the search terms, we found 1875 original papers. We proceeded by sorting out papers that focused on neurological diseases such as Parkinson syndrome, Klein Levine syndrome, brain injuries and Kluyver Bucy syndrome. We also excluded studies that focused on medication side effects, single case studies, studies in animals or studies focusing on bipolar/manic conditions. After this sorting process, 475 studies remained.

We included articles that were (1) published in the last 20 years, (2) were peer-reviewed, (3) were published in English, (4) presented empirical data from humans, (5) assessed Compulsive Sexual Behavior with at least one standardized questionnaire (6) and included at least 10% of females and males in their population.

**Figure 1:**
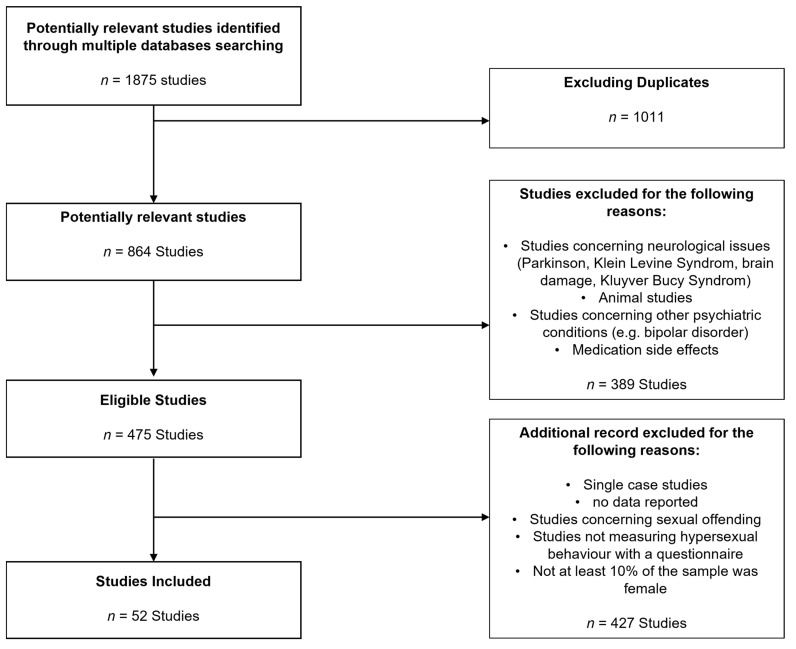
Preferred Reporting Items for Systematic Reviews Flow Diagram (PRISMA).

We identified 52 studies that matched our search criteria and that were relevant to our research question (compare Figure 1). Only studies that targeted women as well as men were included in order to compare both groups. We included studies that focused on psychiatric comorbidities and on underlying psychological problems, such as insecure attachment styles or difficulties in emotional regulation and excluded experimental studies. In the following review, we will use the term CSBD if the compulsive sexual behavior was reported using a questionnaire cut-off (thus indicating clinically relevant compulsive sexual behavior/CSBD) or other indicators such as a formal categorical diagnosis. We use the term Compulsive Sexual Behavior (CSB) for dimensionally reported data.

## 3. Results

Compulsive Sexual Behavior was operationalized via different questionnaires. While a wide variety of measures were used, the Hypersexual Behavior Inventory (HBI) [16] was most common. Other measures included the Hypersexual Disorder Questionnaire (HD) [17], Hypersexual Disorder Screening Inventory (HDSI) [18], Hypersexual Behavior Consequences Scale (HBCS) [19], total sexual outlet (TSO, number of orgasms in the last week), Sexual Addiction Screening Test (SAST) [20], Sexual Compulsivity Scale (SCS) [21] and Compulsive Sexual Behavior Inventory (CSBI-13) [22]. Gender differences in hypersexual behavior have not yet been extensively studied. Even the included studies did not always report comprehensive gender differences. In 22 of the 52 studies, gender differences have been analyzed.

### 3.1. Frequency of Sexual Behavior by Gender

#### 3.1.1. Convenience Sampling

Most studies used online convenience sampling to investigate CSBD and reported higher scores of the CSBD measuring instruments for men compared to women. In some of these convenience samples, the percentage of individuals meeting the CSBD cut-off seemed quite high (e.g., 13.1% of females and 45.4% of males [23]). Comparably high results (19.2% endorsing CSBD (79.1% male and 17.1% female)) were reported in Walton et al. [24] as well as Walton et al. [25] (18.4% of their sample meeting the cut-off; 67% male and 32.9% female).

Lower numbers were reported by Carvalho et al. [26] who reported 6% of males and 3% of females endorsing a single item regarding lack of control of their sexuality. In a subsequent cluster analysis, they reported that 2.8% of their sample fit into a control/consequences cluster (6% men and 3% women). In a cluster analysis, Vaillancourt-Morel et al. [27] reported higher numbers for the compulsive cyberpornography user cluster (11.8%), with men being more likely to belong to this cluster. In a community sample, Castro-Calvo et al. [28] reported 7.8% of individuals meeting the CSBD cut-off (HBI).

CSBD frequencies differed immensely between populations. Especially high frequencies were reported in student populations, with frequencies as high as 27.9% of males and 9.2% of females meeting the HBI cut-off [29]. In a study measuring CSBD with a composite index based on previously validated scales (HBI, SCS and SAST), the number decreased to 10.1% of students (of which 30.6% were female) endorsing CSBD [28]. Nevertheless, the gender effect with higher indices for males was reported in numerous student samples and with a variety of measurements (HDSI, [30]; HD and HBCS [31]; SCS [32,33]).

Another specified sample group consisted of users of geosocial networking apps (GNAs, e.g., Grinder), where more users of these apps met the HBI cut-off compared to non-users (e.g., 7.7% compared to 3.7% [34]). The gender effect of higher scores in men remained in these populations across measurements (e.g., measured with SAST [35]) and was also true for general online dating [36]. Notably, one study on individuals endorsing online dating reported no gender effect on CSB (measured with SAST) even though 35.1% of their sample showed high levels of CSB. In a study on fitness center clients, 2.0% of women and 3.8% of men met clinically relevant HBI scores [37].

By measuring the ratio of female and male individuals with CSBD, several studies reported similar ratios of approximately 30% females (e.g., 15.8% of men and 5.7% of women [29], 27.2% of individuals meeting the CSBD cut-off were female [28] and 33.8% females in the CSBD cluster [38]).

#### 3.1.2. National Surveys

Larger, national studies report fewer individuals with CSBD. In their large sample (*n* = 2325) representative of the USA, Dickenson et al. [13] reported clinically relevant levels of distress/impairment associated with sexual compulsivity for 8.6% of their sample (10.3% men and 7% women).

When investigating individuals who conceded to having watched pornography in the last year, Bőthe et al. [39] identified 7.4% high frequency problematic pornography users in their sample by using Latent Profiles Analysis with different measures including HBI (*n* = 14,006 of which 30% were female). In another Hungarian study (*n* = 18.034, 34.0% females) Bőthe, Bartok et al. [40] reported an effect of gender and sexual orientation on HBI scores: LGBTQ males had the highest HBI scores and heterosexual females had the lowest scores. LGBTQ females and heterosexual males showed a more complex picture: LGBTQ females scored higher on the coping aspect of CSB whereas heterosexual males scored higher on the consequences.

#### 3.1.3. Clinical Samples

Comparable to convenience samples and national samples, clinical sampling amounted to similar gender effects on the CSBD measures. In a clinical outpatient sample, Fuss et al. [41] reported an albeit smaller gender effect. They investigated CSBD in 539 adult outpatients with OCD (51.8% females) with expert interviews. The lifetime prevalence of CSBD was 5.6% and, for current CSBD, is 3.3%. Both incidences were significantly higher in males than in females (lifetime: 8.8%/2.5% and current 5.4%/1.4%).

In a study comparing CSBD in adults with Autism Spectrum Disorder (ASD) and healthy controls (HC), Schöttle et al. [42] reported that 30.4% of ASD males met the HBI cut-off for CSBD, whereas only 10.0% of ASD females met this cut-off [42].

Two studies investigated CSBD in participants with substance dependence [43,44] who also reported very high CSBD frequency. Diehl et al. [43], reported 28.4% of participants with substance dependence displayed clinically relevant levels of CSBD (measured with SAST). Likewise, Antonio et al. [44] reported that 18.9% of participants with alcohol dependence and even 40.3% with crack/cocaine dependence showed clinically relevant levels of CSBD (SAST).

In samples specifically targeting CSBD populations, the picture seems inconclusive. In a DSM-5 field trial, Reid et al. [17] reported mainly men (94.7%) being referred for CSBD treatment. In contrast to these findings, Görts Öberg et al. [15] investigated a clinical sample of individuals seeking help for CSBD. Fifty percent of their sample met the HDSI cut-off, with a higher prevalence among women (75%) compared to men (45%). They found that women had higher scores on the HDSI, reported more sexual activity with consenting adults and were more concerned about physical injuries and pain.

### 3.2. Gender Differences in Psychological Traits and Psychiatric Comorbidity

In an attempt to further conceptualize CSB, many studies regarding psychiatric comorbidities and psychological traits have been conducted. In the studies reviewed CSB has been reported to have correlations with perfectionism [45], paraphilias and sexual dysfunction [30,46], mood disorders (e.g., anxiety and depression) [26,35,38,41,47,48], experience of childhood sexual abuse [47,49,50], OCD [35,41], Autism Spectrum Disorder [42], general emotional dysregulation [31], substance abuse [43,44], maladaptive schemata [51], excitement seeking in general [45,52], compulsivity and impulsivity [47,49,53], high sexual arousal/excitation [25], higher neuroticism [47], avoidant coping mechanisms [54], high rates of psychiatric comorbidity in general [38], high distress rates and negative emotions [14], boredom [48], general childhood adversities and trauma [30,33,43,49,55,56,57,58,59], pathological buying [37], insecure attachment style [60,61,62] and risky sexual behavior [32] in both genders.

Gender differences have not been studied extensively in the reviewed studies. Twenty-two studies calculated at least some analyses to compare men and women. As the studies are mostly of a correlative nature, a variety of correlative gender differences were found.

Of the reviewed studies, four studies focused on sexual behavior [52,63] and paraphilic interests [23,30] and reported some gender effects. For example, Rettenberger et al. investigated a large online sample (*n* = 1749, 56.5% female) and reported lower scores on sexual excitation (SES) and CSB (HBI) for women and consequently higher scores on sexual inhibition dimensions as well. Interestingly, CSB seemed connected to sexual excitation, “fun-seeking”, low scores on threat performance consequences and conscientiousness for both genders [52].

In contrast to this finding, Engel et al. [23] reported some gender effects regarding sexual behavior correlations. For women, CSB was associated with partnered sexual activity, TSO-masturbation and time of pornography consumption, while there were no significant correlations between CSB symptom severity and partnered sexual activity in men. However, there were significant but weaker correlations between pornography consumption and TSO-masturbation. When compared to women, men had higher rates of pornography consumption (e.g., 6.64 h weekly in men compared to 1.05 h in women; d = 0.41) and elevated rates of problematic cybersex (d = 0.96) [23]. This ties in with the finding of Bőthe et al. who reported a moderate positive association between CSB and Problematic Pornography Use (PPU) in women but a strong association in men [64]. In a second study, Bőthe et al. reported a greater relationship between impulsivity and CSB (β = 0.41 and β = 0.42) than between CSB and compulsivity (β = 0.21 and β = 0.16) among men and women, respectively [53]. In a recent study by Efrati et al. [63], CSB was connected to engaging only in online sexual activities and female gender in an adolescent sample.

When investigating the association between CSB, paraphilic behaviors and sexual functioning, gender differences concerning coercive fantasies and behavior [23] and general paraphilic interests [30] were reported. Paraphilic interests were also associated with CSB symptom severity in a study with Italian university students, with higher HDSI scores in all paraphilic interest groups (except transvestitism). Additionally, gender differences relative to the nature of paraphilic interests were reported: men exhibited higher prevalence of exhibitionism, sadism, voyeurism and frotteurism, while women exhibited higher prevalence of masochism and fetishism [30]. In accordance with this finding, coercive fantasies were found to be more prevalent in men compared to women (*p* < 0.001), as was the past behavior of forcing someone to perform sexual behaviors (*p* < 0.001) or using child abuse images (*p* < 0.001) [23]. When compared with the general population, women with CSB reported more fantasies about forced sexual actions as well as actual past behavior of forcing someone to perform sexual acts. The same was true for men [23].

Another focus of past research was psychiatric comorbidity with 10 studies reporting gender effects on some psychiatric correlates, such as depression [23,28,35], anxiety [28,35], OCD [35,41], Autism Spectrum Disorder (ASD) [42], ADHD symptoms [64], sexual dysfunction [30,46] and general stress proneness [15,65].

Regarding depression and OCD, the picture seems relatively consistent: In all four studies [23,28,35,41], depressive symptoms, anxiety and OCD symptoms were more strongly associated with CSB symptom severity in men than compared to women. For example, Levi et al. investigated the impact of compulsivity, depression, anxiety and impulsivity on problematic online sexual activities in individuals who use the internet for finding sexual partners. In their sample, OCD, depression and anxiety contributed more to CSB in men (40%) than in women (20%) [35].

In regard to ASD, Schöttle et al. [42] measured CSB in 96 individuals with ASD (58% male) and 96 matched healthy controls. They reported that ASD patients had a higher HBI sum score than healthy controls and that more ASD individuals met the HBI cut-off. They found that men with ASD reported more CSB than their healthy control counterparts, while there was no such difference in females.

Bőthe et al. found gender differences in the association between ADHD symptoms and Problematic Pornography Use (PPU): They found a moderate association for men but a weak association for women [64]. The authors conclude that for men with ADHD, pornography use could be some kind of self-medication, whereas women with ADHD symptoms do not use pornography but other sexual behaviors instead. This could be due to a more normative approach to pornography among men. Two studies found an association between CSB in response to stress [15,65], with women reporting greater stress vulnerability. Sexual dysfunctions were associated with CSB in both genders [30]. Furthermore, Burri et al. reported that women with higher sexual compulsivity (measured with SCS) reported worse sexual functioning [46].

Another branch of correlative studies was conducted in the realm of personality traits, with three studies reporting gender differences on personality traits in individuals with CSB [26,36,65]. For example, Shimoni et al. conducted an analysis of variance and found that personality factors contributed significantly to the variance of CSB by explaining as much as 11.7% of the variance. Interestingly, in their study, greater neuroticism was indicative of CSB in men but not women [36]. In contrast to this finding, Carvalho et al. reported from their cluster analysis that women with higher neuroticism were more likely to be sorted into a “control cluster”, which is indicative of CSB [26]. When studying gender differences in a clinical population, Reid et al. [65] found no gender differences in the NEO domain neuroticism, but in the domains conscientiousness (competence: w < m; dutifulness: w < m; achievement striving: w < m) and impulsivity (w > m in excitement-seeking), thus indicating that conscientiousness seems more relevant for men, while impulsivity seems to be a more relevant factor for women with CSBD.

Often discussed antecedents to CSB include childhood adversities and trauma, which were investigated in ten of the reviewed studies [30,33,43,49,55,56,57,58,59] of which six investigated gender differences. One study [30] found a correlation between the HDSI and a history of sexual abuse (β = 0.144 and *p* = 0.001) in women but not in men. Similarly, in a drug user outpatient group, Diehl et al. [43] found that women had higher childhood maltreatment rates than men (sexual abuse: OR: 2.9; physical abuse: OR: 3.7). In contrast to this finding, Slavin et al. [49] found an interaction between Childhood Sexual Abuse and Adolescent/Adult Sexual Abuse (CSA/AASA) and gender. In this sample, CSA/AASA impacted CSB in men more strongly than in women. Likewise, Fontanesi et al. [55] reported an effect of post-traumatic stress symptoms on CSB with the male gender being a risk factor in the development of CSB. In contrast to these findings, some studies reported no gender effects. While Meyer et al. [56] reported a correlation of CSB (measured with SAST) and sexual abuse (r = 0.26 **) in their sample (812 individuals, 62% females), they did not find any gender effects. Similarly, Vaillancourt et al. [58,59] reported a correlation of CSB and CSA frequency without gender effects.

One study used exploratory factor analysis to investigate the latent structure of CSB in men and women. This study concluded that CSB in males seemed to be more consistent with a taxon rather than with a continuum. Female CSB on the other hand seemed to be more dimensional and showed evidence of continuous latent structure [29].

## 4. Discussion

### 4.1. CSB-Frequency by Gender

With the introduction of CSBD into the ICD-11, the interest in research has sparked in this topic [66]. Up until now, CSBD has been measured with a variety of questionnaires and indices. In the studies reviewed for this article, the HBI [16] was used most often, followed by the SCS [21], HBCS [17], HDSI [18] and SAST [20]. The variety of indices reflects the debate on the different concepts that were used in the past to describe CSBD (e.g., addiction concept, compulsive behavior, impulsive behavior) (compare [2,66]). The different indices are one possible explanation for the wide range (2.8% up until 35.1% in convenience samples; 3.3% up until 8.3% in national surveys; 3.3% up until 40.3% in clinical samples) of estimated prevalence.

While studies using convenience or online sampling include more women in the last years, studies with clinical samples do still focus more strongly on men. In convenience samples, men show higher scores in indices measuring CSB. This is true for a wide variety of measures, although the variety makes it harder to compare results. It seems quite stringent that men who score higher in measures of CSB also report more masturbation frequency and more sexual behaviors than women. The same is true for sexual behavior, especially masturbation, in men in the general population [67].

CSB rates seem especially high in student populations. For example, in their university student sample, Castro-Calvo et al. [28] found rates of 10.1% of CSBD out of which about 30.0% were female. Comparably, Kingston et al. [29] reported 9.2% of women in their university sample meeting the HBI cut-off. Similarly, even up to 19.4% of students met CSBD criteria measured with the HD and HBCS [31]. We only found one study that did not report a gender difference for the measure in CSBD [48] measured with the SAST.

At the same time, the picture seems inconclusive regarding clinical samples. Clinical samples mostly target other clinical populations and survey CSBD as a correlate. Rates of CSBD have been investigated in samples of patients with OCD [41], ASD [42] and those with alcohol and drug associated disorders [43,44]. While prevalence in the OCD sample was comparable to prevalence found in large non-clinical samples (5.4% in men and 1.4% in women [41]), prevalence seemed to be much higher in individuals using drugs [43,44]. It is important to note that the SAST was used in the drug-using sample using a cut-off of six, which is validated for the Brazilian population. In other samples the cut-off of 13 is usually used. Still, this makes the numbers harder to compare.

In this context, it is also important to mention that high frequency sexual behavior is not identical with CSB or CSBD. Bőthe et al. [39] found in their study that individuals who were in the high frequency sexual behavior group (in this case pornography consumption) differ from people with high frequency problematic sexual behavior. These groups were distinguishable according to their HBI scores, which verifies that there is such a thing as unproblematic high frequency pornography consumption and that it is not to be confused with CSBD. Vaillancourt-Morel [27] reported a similar finding in their study by using the SCS scores to differentiate between clusters (recreational pornography use, highly distressed non-compulsive use and compulsive use).

### 4.2. Gender Differences in Comorbidities and Correlations

New models of CSBD are currently emerging (e.g., the Sexhavior Cycle of Hypersexuality [68], the Moral Incongruence Model [66] and the Integrated Model of CSBD [2]) and it is important to test these models and their assumptions. In these models, gender differences regarding sexual response are not explicitly addressed. They are mostly addressed as different psychosocial influences on women and men.

In our review, 22 studies reported at least some analyses comparing men and women. As the studies are mostly of a correlative nature up to now, a variety of correlative gender differences were found in the study.

#### 4.2.1. Psychological Traits and Psychiatric Comorbidity

There were two studies that reported higher rates of neuroticism in women than compared to men [26,36]. Women also reported greater stress vulnerability [65]. On the other hand, high symptoms of depression were more strongly associated with high levels of CSB in men when compared to women in one online study [23]. Görts Öberg et al. [15] even found an association between CSB in response to stress, especially in women. Women expressed more issues with trust in one study [36]. Higher neuroticism and stress vulnerability seem to be a pathway that could explain CSB in women, whereas other influencing factors seem to be more prominent for men, e.g., ADHD [64], ASD [42] or OCD, depression and anxiety [35]. When applied to the Integrated Model of CSBD [2], this could mean that neuroticism, stress vulnerability and trust issues could either mediate psychiatric comorbidity or could facilitate using sex as a coping mechanism by themselves, thus strengthening the excitatory pathway.

According to a student sample, women had lower scores regarding sexual excitation as well as higher sexual inhibition scores, especially in the realm of consequences [52]. Lower scores of feeling threatened by consequences of sexual behavior corresponded to higher scores on the HBI [52]. In other words, the less people are concerned with the consequences of their (sexual) actions, the easier it is to display (compulsive) sexual behavior. However, they did not find a moderation effect of gender in their study. This seems to be at odds with the finding that women scored higher on inhibitory scales and lower on exhibitory scales compared to men in the general population [69]. It would seem plausible that similar effects would be observable in the CSBD population. The absence of the gender effect could indicate that the gender differences become less pronounced as soon as individuals engage in compulsive sexual behavior. On the other hand, in a Swedish study [15], women reported feeling more threatened by physical consequences (=inhibitory factor) of their sexual behavior (e.g., becoming a victim of violence but also unwanted pregnancies) compared to men. Speculatively, the need to act on compulsive sexual behaviors becomes so urgent that women will disregard inhibitory influences.

#### 4.2.2. Specified Sexual Behaviors

Some studies showed a connection of CSB to unusual sexual behaviors and sexual dysfunctions. In their sample recruited from sex addiction help forums, Engel et al. [23] found a high prevalence of coercive fantasies for both genders, but men had more often engaged in sexually coercive behavior. In this study, levels of CSB severity were also associated with sexual fantasies involving coercion and actual coercive behavior [23]. CSB seems to be connected to paraphilic behaviors as well, with men reporting higher prevalence of exhibitionism, sadism, voyeurism and frotteurism, whereas women had higher rates of masochism and fetishism [30]. Sexual dysfunctions and general psychopathology were associated with CSB in their sample. These findings indicate that men as well as women with CSB report paraphilic fantasies and behaviors, but women tend to report paraphilias which potentially could pose more threat to themselves (e.g., masochism when not practiced in safe boundaries) and men report paraphilias with more potential for harm for others (e.g., coercive behavior, frotteurism and non-consensual sadism).

Interestingly, consuming pornography seems to be less important for women suffering from CSBD compared to men [28,70]. Masturbation and consumption of pornography are relatively low risk sexual behaviors other than them being more normative for men than for women.

#### 4.2.3. Childhood Adversities

One often-discussed pathway in the development of CSB is via experiences of childhood adversities or a history of sexual abuse [30,33,43,49,55,56,57,58,59]. The picture on if and how childhood adversities affect genders differently is still inconclusive. One study found a correlation between CSB (measured with the HDSI) and fatherly neglect in women but not in men. The same was true for a history of sexual abuse [30]. While severe sexual abuse is more prevalent for females [71] in the general population, one study [49] found that a history of childhood and adolescent sexual abuse affected CSB in men more strongly than in women. In their review, Slavin et al. [72] found that gender differences regarding the link between CSB and childhood sexual abuse were inconclusive. More data are needed to investigate this link and possible gender differences further. As there has been some research already conducted on the connection between CSA and CSB, data still remain sparse on other childhood adversities.

As distress is a unique subjective feeling for the individual, some researchers note that at least some portion of distress can be attributed to moral incongruence. The Moral Incongruence Model [73] sets a focus on perceived problems with pornography via two pathways: moral incongruence and dysregulation. While dysregulation can itself result in distress, they argue that a sizable portion of perceived problems with pornography is explained via the Moral Incongruence pathway: Religiousness might result in moral disapproval of pornography. When individuals use pornography anyway, they might experience moral incongruence and following distress. This takes into account that in clinical settings people seek help for sexual behavior that does not seem excessive but causes distress for the person [73]. What still needs to be explored is the question of how moral incongruence affects men and women differently. Women could be exposed to more moral incongruence because normative female sexual behavior is still seen as having lower sexual excitation and higher sexual inhibition [74]. When a female with high levels of sexual activity compares themselves to their normative view of other women, they could experience distress and falsely classify themselves with CSB [23].

Notably, the ICD-11 diagnostic guidelines specifically exclude distress that is “entirely related to moral judgements and disapproval about sexual impulses” (ICD−11 for Mortality and Morbidity Statistics’; quoted according to [2]). Therefore, individuals being distressed due to moral incongruence only do not fulfill the diagnosis of CSBD. Distinguishing what part of distress is due to moral incongruence and at what point the individual displays CSB or CSBD will still be an ongoing debate among clinicians. In a recent study, most mental health professionals were able to correctly diagnose CSBD in the ICD-11 trials [75], thus being able to reject diagnosis for individuals who were distressed due to moral incongruence.

### 4.3. Future Implications

In a DSM-IV trial [17], Reid et al. found higher scores on the HBI in men. On the other hand, Öberg Görts et al. [15] demonstrated higher burden on women. In our review, almost all studies reported higher scores in CSBD indices in men. It is possible, that women only seek help when they experience their behavior as threatening to their private and professional lives, thus waiting longer to reach out to an outpatient clinic. Possibly, it is easier for men to report their behaviors because high frequency sexual behaviors are culturally more expected from men [69], which results in the over-reporting of sexual behaviors in men and underreporting of sexual behaviors in women (“sexual double standard”) [76]. This might concern the CSBD population as well. Here, it is to be discussed if it is helpful to use the same cut-off scores on indices for men and women. Due to the sexual double standard [76] and generally higher frequency of sexual behavior in men, it is possible that current measurements will result in the underreporting of Compulsive Sexual Behavior in women when we measure it the same manner as in men (compare [77]). Women are still especially scarce in clinical studies, which limits the clinical implications of these findings. Additionally, as questionnaire data are used dimensionally in many studies, the implications for individuals with diagnosed CSBD are also limited.

With the inclusion of CSBD in ICD-11, there is hope that researchers will start using comparable questionnaires and cut-offs in their future studies. In addition, the release of binding diagnostic categories will verify that the same construct is being investigated. Another possible development will be that CSBD will be more prevalent because mental health professionals will be able to use the diagnosis to treat their patients. For this, reliable therapy options are still required. As we have demonstrated that CSB seems to have at least somewhat different expressions in men and women, it is recommended that gender specific treatment foci should be implemented.

As there are some indications that women with CSB have other concerns than men with these behaviors (e.g., they worry more about STIs and pain), researchers and clinicians should investigate the needs and concerns of women even if they seem to be less often affected by CSBD. It might be useful to explicitly ask for sexual behaviors (and also compulsive sexual behaviors) when women present themselves for other disorders that pertain to impulse control, substance abuse or mood disorders. On the other hand, it seems to be advisable to ask about paraphilic fantasies in men as well as women.
